# Retraction Note: Clinical characterization of familial 1p36.3 microduplication

**DOI:** 10.1007/s10048-023-00735-7

**Published:** 2023-10-07

**Authors:** Junping Jiao, Yuping Wang, Yue Hou, Chao Gao, Huimin Shi, Shujuan Tian

**Affiliations:** 1 Department of Neurology, The First Hospital of Hebei Medical University, Shijiangzhuang City, Hebei Province China; 2 Xuanwu Hospital Capital Medical University, Beijing, China

**Retraction Note: Neurogenetics (2023) 24:201-208**

10.1007/s10048-023-00722-y

The Editors in Chief have retracted this article due to substantial overlaps with previously-published text by different authors [1, 2]. However, the overlaps do not concern the data reported in the article, and due to the unique nature of this data, the authors have been offered the option to submit a revised manuscript for further peer review. Junping Jiao, Yuping Wang, Yue Hou, Huimin Shi and Shujuan Tian agree to this retraction. Chao Gao did not respond to correspondence from the Editors about this retraction.
